# Editorial: Continuing professional development

**DOI:** 10.3389/fmed.2025.1719059

**Published:** 2025-11-20

**Authors:** Ana Da Silva, Ray Samuriwo, Manuel João Costa

**Affiliations:** 1 Faculty of Medicine, Health and Life Science, School of Medicine, Swansea University, Swansea, United Kingdom; 2 School of Health and Social Care, Edinburgh Napier University, Edinburgh, United Kingdom; 3 School of Medicine, University of Minho, Braga, Portugal

**Keywords:** health professions education, continuing professional development, CPD, faculty development, evidence-based practice

## Introduction

The WHO (1) has long maintained that healthcare professional education is integral to the delivery of safe high quality patient care, that is delivered in an equitable, just, and sustainable manner. Continuing Professional Development (CPD) is a vital aspect of healthcare professional education that helps to continually improve the quality and safety of patient care (1–3). This aligns with the view that CPD translates scientific discovery into better care for populations (4). Given the incessant pace of innovation, powered by digital technologies and artificial intelligence, there is an urgent imperative to consider the nature of CPD as a pillar of health professions education.

This Research Topic explores the developments, innovations, and research shaping the future of CPD. In these nine articles from different countries, we characterize four overarching themes: (1) New horizons; (2) Design and Implementation; (3) Context; and (4) Data informed CPD. As a group, these articles demonstrate the evolution of CPD across the world, driven by how scholars and researchers with astute curiosity undertaking work with methodological rigor to drive forward improvement. We invite you to explore the articles within this Research Topic in greater detail and join your voice to this scholarly conversation.

## New horizons

Three articles (Guo et al., Leyland et al., Womack-Adams et al.) bring innovative perspectives from other fields to enrich CPD. One experimental study (Guo et al.) combines humanities into simulation practices in emergency training. This study (Guo et al.) revealed that the observation group (*n* = 40), which participated in a simulation integrated with medical humanities, significantly outperformed the control group (*n* = 39) in both assessment scores and satisfaction levels, highlighting the positive impact of incorporating humanities into simulation training. These results support the necessity for a paradigm shift in continuing professional development (CPD), emphasizing a holistic, competence-based approach rather than focusing solely on skills. Another study (Leyland et al.) in this theme uses Ways of Thinking and Practicing (WTP) (10), an analytic lens from undergraduate biology, to explore medical students' reflections. Although used in the medical student context, WTP brings insights into students' preparedness for practice relevant for CPD, and WTP could also be of value in exploring CPD-related reflections.

The final article in this theme focuses on micro-credentials (Womack-Adams et al.); these are portable credentials that are widely used in other areas of vocational education. This research (Womack-Adams et al.) brings a much-needed rapid review of the use of micro-credentials in CPD across health professions education. A total of 11 articles out of an initial search of 437 were included in the final review, with the majority focused on one profession (*n* = 7, 64%), with nursing being the most commonly discussed health profession (*n* = 5, 45%) (Womack-Adams et al.). The authors (Womack-Adams et al.) call for increase in consistent terminology and coordination on a broader scale in this area. In summary, these three research pieces open the door for a conversation on CPD that drives its impact from the integration of multiple disciplines, multiple theoretical perspectives, and learning practices from other fields of education.

## Design and implementation

In the past, too much CPD has focused on what—new drugs, guidelines—rather than the show of learning (5, 6). If the aim is improved patient outcomes, the how matters at least as much as the what. Two studies (Yingxia et al., Mueller et al.) in this theme do exactly that, in different ways, bringing our attention to the fact that how we design CPD matters for its efficacy both from an individual and wider systems perspective. Yingxia et al.'s literature and policy mini-review emphasizes that post-competency types of training for post-registration nursing and its impact on transition to practice; concluding that implementation and evaluation are critical to success. Mueller et al. compares two commonly used modalities of delivery of CME/CPD—in-person vs. livestream. Participants showed significant improvements in post-COVID knowledge (47% correct pre-course to 54% correct post-course, *p* = 0.004) but did not differ significantly between in-person vs. livestreamed sessions (Mueller et al.). Given the results, the authors (Mueller et al.) suggest that distance learning, which is less resource-intensive and has no detrimental impact on quality, may be a feasible option for content delivery.

## Context

Three articles (Edmealem et al., Suleiman, Al-Haqan et al.) in this Research Topic demonstrate the importance of considering the various contexts in which CPD operates: professional, geographical, regulatory. These articles are methodologically varied and address different questions; however, all demonstrate how important it is to consider the context to ensure research findings are both valid and relevant for practice. Edmealem et al. conducted a systematic review and meta-analysis of 11 studies researching professionalism in nursing in Ethiopia between 2004 and 2024. The definitions of professionalism used demonstrate well how being attuned to the context is important even in complex synthesis works. Focusing their review on the Ethiopian perspective offered important insights for nursing in Ethiopia and for the scholarly conversation on CPD and professionalism globally. Suleiman's study demonstrates that adverse drug reaction (ADR) underreporting in Jordan is impacted by factors that can be addressed with CPD. This study paves the way for the design of CPD interventions that are contextually relevant and evidence-based, linking both the call for context in CPD and its call for evidence-based. Finally, Al-Haqan et al. developed and validated the Kuwait Advanced Competency Framework (KACF), based on the International Pharmaceutical Federation (FIP) Global Advanced Development Framework (GADF). The GADF is a validated tool intended to support the professional development and recognition of the pharmacy workforce globally (7). This study is an excellent example of how global frameworks can be adapted to identify gaps in local CPD contexts, while using global standards as scaffolding for national competency development.

## Data informed CPD

Data-driven CPD is a concept that has been part of the conversation for a while (8). In this Research Topic, Pizzuti provides a perspective article that offers an invigorating view on what could be achieved. This article presents a view of how electronic health records could hold the key to developing adaptive CPD that responds to the real needs of healthcare systems, based on data from practice and education systems.

## Conclusion

This Research Topic contributes to previous conversations on how to reimagine CPD (5, 9). Here we bring together contributions that examine the how; elevate context; operationalise evidence-based and data-driven approaches; and broaden the horizons, shifting paradigms and theoretical lenses. As the challenges and opportunities brought to us by for example, Artificial Intelligence and new technologies lie ahead, the research here presented reflects a field rigor in its methods and forward-thinking in its approach.

The task now is collective: to design, deliver, embed, evaluate, and sustain CPD that translates learning into better practice and improves patient outcomes. As we bring this Research Topic to a close, we encourage continued dialogue to interrogate assumptions and to move toward embracing new paradigms that are evidence-based, contextual, and oriented toward outcomes.
